# Ultra-thin freestanding graphene films for efficient thermal insulation and electromagnetic interference shielding

**DOI:** 10.1039/d3ra00638g

**Published:** 2023-06-27

**Authors:** Peng Zhang, Zhi Cao, Chunle Liu, Pengni Li, Hui Kong, Ting Li, Xiaomin Luo, Jianyan Feng, Kaiyun Yuan, Ruqing Xu

**Affiliations:** a College of Bioresources Chemical and Materials Engineering, Shaanxi University of Science & Technology Wei Yang District Xi'an 710021 Shaanxi China luoxiaomin@sust.edu.cn fengjianyan@sust.edu.cn; b National Demonstration Center for Experimental Light Chemistry Engineering Education, Shaanxi University of Science &Technology Wei Yang District Xi'an 710021 Shaanxi China; c Shandong Taikai Power Electronic Co., Ltd. Taian 27100 Shandong China; d Tongxiang Quality and Technology Supervision Center Tongxiang 314599 Zhejiang China; e Zhejiang Zhanyu New Materials Co., Ltd Quzhou 324400 Zhejiang China

## Abstract

The preparation of freestanding graphene films by convenient and environmentally friendly preparation methods is still the focus of attention in various industrial fields. Here, we first select electrical conductivity, yield and defectivity as evaluation indicators and systematically explore the factors affecting the preparation of high-performance graphene by electrochemical exfoliation, then further post-process it under volume-limited conditions by microwave reduction. Finally, we obtained a self-supporting graphene film with an irregular interlayer structure but excellent performance. It is found that the electrolyte is ammonium sulfate, the concentration is 0.2 M, the voltage is 8 V, and the pH is 11, which were the optimal conditions for preparing low-oxidation graphene. The square resistance of the EG was 1.6 Ω sq^−1^, and the yield could be 65%. In addition, electrical conductivity and joule heat were significantly improved after microwave post-processing, especially its electromagnetic shielding performance with a shielding coefficient of 53 dB able to be achieved. At the same time, the thermal conductivity is as low as 0.05 W m^−1^ K^−1^. The mechanism for the improvement of electromagnetic shielding performance is that (1) microwave reduction effectively enhances the conductivity of the graphene sheet overlapping network; (2) the gas generated by the instantaneous high temperature causes a large number of void structures between the graphene layers, and the irregular interlayer stacking structure makes the reflective surface more disordered, thereby prolonging the reflection path of electromagnetic waves among layers. In summary, this simple and environmentally friendly preparation strategy has good practical application prospects for graphene film products in flexible wearables, intelligent electronic devices, and electromagnetic wave protection.

## Abstract 2

The preparation of freestanding graphene films by convenient and environmentally friendly preparation methods is still the focus of attention in various industrial fields.

## Graphical abstract

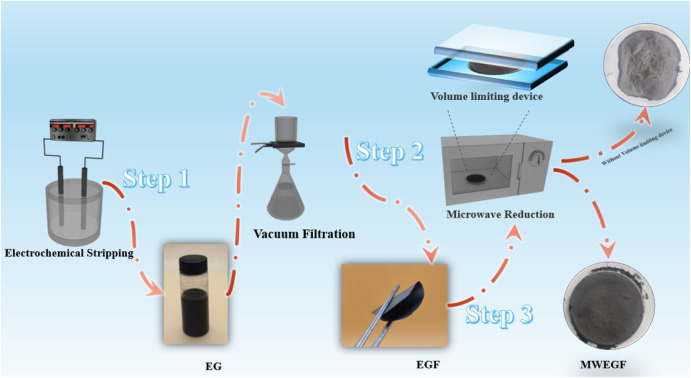

## 1. Introduction

With the rise and commercialization of modern information technology, especially 5G technology, electromagnetic waves, as a medium for disseminating information, have brought convenience to people's production and life in sophisticated electronics, cell phones, and computers.^1^ However, electromagnetic pollution has become an urgent environmental problem to be solved. At present, most electromagnetic radiation pollution from systems such as microwave communication, communication base stations, and high-voltage transmission lines is caused by human factors. Electromagnetic radiation not only causes a disturbance or even damage to the normal working of electronic equipment, but its harmful effects on human health are becoming increasingly prominent.^2^

Graphene-based film materials have good prospects for practical applications in electronics, especially in the field of electromagnetic shielding, occupying an irreplaceable position due to their unique interlayer stacking structure, as well as their good electrical conductivity and ultra-lightness.^3^ The multi-layer reflective interface and electron transfer properties make the research on the construction, assembly and modification of graphene-based films a current research hotspot. The physicochemical properties of individual graphene nanosheets often directly affect the stability of their dispersion, the homogeneity of the assemblies, the integrity of the conductive network and the orderliness of the stacked structure.^4–6^ Graphene nanosheets are irregularly shaped, which is not conducive to the ordered stacking of film formation. Introducing block polymers, cross-linkers, and nanomaterials into the interlayer of graphene has achieved remarkable results in macroscopic graphene films. Although the dispersion of graphene can be significantly improved, the introduced impurity ions tend to degrade the electrical, thermal, and magnetic properties of graphene films.^7–9^ In addition, the traditional preparation methods, such as the redox method, mechanical exfoliation method, and liquid deposition method,^10–12^ have insurmountable problems such as being time-consuming, easy environmental pollution and performance defects caused by hazardous chemicals. The chemical vapour deposition method^13^ is a complex preparation process. Expensive equipment requires harsh environments such as high temperatures and high vacuum, which need to transfer 2D materials from the metal surface to the target. The manufacturing process is difficult to control and even introduces impurities or defects into 2D materials, resulting in decreased product performance and difficulty removing graphene from the substrate. Therefore, convenient graphene preparation conditions and tunable graphene products are essential for constructing graphene thin films.

Electrochemical exfoliation is a top-down graphene preparation method, which has the advantages of simple operation and environmental friendliness (using ionic liquids, electrolytes, water or surfactants) and does not require volatile solvents or reducing agents that can form highly controllable flakes.^14^ That can be quickly industrially produced using known electrochemical cell design and engineering principles. Various graphite materials, such as graphite flakes, graphite rods, and high-oriented pyrolytic graphite (HOPG), can be used as the carbon source for the electrochemical preparation of graphene.^15–17^ There are still a tiny amount of oxygen-containing groups on the graphene prepared by electrochemical exfoliation, which usually weakens graphene's electrical conductivity, thermal conductivity, and electromagnetic shielding properties. Many factors affect graphene's electrochemical exfoliation (defect degree, rate, electric field parameters, equipment, *etc.*). Zhang *et al.*^18^ improved the electrochemical exfoliation efficiency by deploying a method containing organic solvents and cations. Tetrabutylammonium cations in propylene carbonate can intercalate into graphite efficiently under reducing conditions. The product showed an ultra-low defect degree of 0.05 (*I*_D_/*I*_G_). Justin Raj *et al.*^19^ introduced ultrasonic equipment simultaneously as electrochemical exfoliation. The pulse (2/4) of 2 s sonication and 4 s rest time can be used in acidic, neutral and alkaline conditions. Both of them promote the electrochemical exfoliation process; Lei *et al.*^20^ explored the effect of high and low voltage on the oxidation degree of graphene, and they evidenced two different regimes during the synthesis by Raman and XPS analysis: slow kinetics of exfoliation at low voltage *vs.* high concentration of OH˙ radicals at high voltage. Recently, Vaiva Nagyte^21^ conducted a systematic study of the Raman spectrum of electrochemically exfoliated graphene, produced using different electrolytes and types of solvents in varying amounts. All in all, it is urgent and significant to explore the preparation scheme of graphene with the advantages of easy industrial production, economy, simplicity and environmental friendliness.

Here, graphene was prepared by the electrochemical exfoliation method. The effects of electrolyte type, concentration, voltage and pH value on the properties of graphene prepared by electrochemical exfoliation were discussed in detail, with conductivity, yield and defect degree as evaluation indicators. The optimum conditions for the electrochemical exfoliation of graphene with double electrodes were obtained. At the same time, the self-supported graphene films were treated by microwave reduction under volume-limited conditions. The conductivity, thermal conductivity and electromagnetic shielding performance of graphene films before and after microwave reduction were compared and discussed.

## 2. Experiment

### 2.1. Reagents

Graphite (99% C content), chemically pure, Tianjin SKF Co., Ltd.; ammonium sulfate, sulfuric acid, analytical grade, Tianjin Tianli Chemical Reagent Co., Ltd.; ammonium chloride, sodium sulfate, diammonium hydrogen phosphate, tripolyphosphoric acid sodium, analytically pure, Xi'an Chemical Reagent Factory; potassium sulfate, analytically pure, Tianjin Fuyu Fine Chemical Co., Ltd.; sodium hydroxide, analytically pure, Tianjin Hedong District Hongyan Reagent Factory; ultrapure water, self-made in the laboratory.

### 2.2. Preparation of highly conductive self-supporting graphene film (EGF)

The EG dispersion liquid was freeze-dried into powder and ultrasonically dispersed in 50 mL of deionised water. The EG dispersion liquid was filtered on a polytetrafluoroethylene membrane with a Buchner funnel, dried in an oven for 4 hours after manually peeling off the substrate, and taken out to obtain a self-supporting graphene film (EGF).

### 2.3. Preparation of highly conductive self-supporting electrical graphene membranes by microwave reduction (MWEGF)

**Fig. 1:**
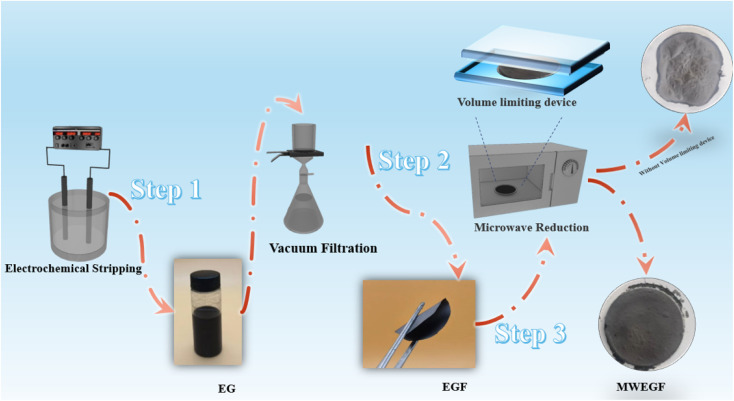
Schematic illustration of the preparation of EG, EGF and MWEGF.

The prepared EGF was laid flat on a 5 mm thick quartz plate, covered with another 5 mm thick plate and fixed. Then put them in the microwave oven, adjust the power to medium, and set the timer to one minute. Then it was taken out to obtain microwave-reduced graphene films (MWEGF). The schematic diagram of MWEGF preparation is shown in Fig. 1.

### 2.4. Structural characterisation and performance analysis

#### 2.4.1. Morphological and structural characterisation

The EG, EGF and MWEGF microstructures were observed by scanning electron microscopy (SEM, model FEI Verios scanning electron microscope, FEI, USA) with an accelerating voltage of 20 kV. The layer structure of EG was observed by transmission electron microscope (TEM, H-600 transmission electron microscope, Hitachi, Japan). The accelerating voltage is 200 kV, and the maximum magnification is 200 000 times. An atomic force microscope (AFM, SPA400-SPI3800 nuclear force microscope, Japan Seiko) was used to test its surface morphology and size in tapping mode. The X-ray diffraction (XRD) test of the sample was carried out using an X-ray diffractometer (model D8 Advance, Bruker, Germany), using D/max 2200 PC Cu target Kα radiation, 2*θ* = 5–40°, and a scan rate of 10° min^−1^. The sample's infrared (FT-IR) spectrum was obtained by a Fourier transform infrared spectrometer (model Vectory-22, Bruker, Germany), and the test wavelength range was 4000–400 cm^−1^. X-ray photoelectron spectroscopy (AXIS ultra DLD X-ray photoelectron spectrometer, Tsushima/Kratos, Japan, XPS) was used to test the sample's composition. A single-frequency Al Kα (1486.6 eV) ray source was used with a power of 250 W. The pollution carbon peak at 284.8 eV is the calibration standard, and the background vacuum is greater than 10–7 Pa. The structural defects of EG were tested with a Renishaw inVia microscopic confocal laser Raman spectrometer (Renishaw, Raman, UK). The incident light wavelength was 325 nm, and the test range was 500–3500 cm^−1^.

#### 2.4.2. Calculation of EG yield

The stripped product was ultrasonic, dialysed, and centrifuged at 3000 rpm for 10 min to disperse and purify the unexfoliated particles. The upper-layer solution and the lower-layer precipitate were freeze-dried and weighed, respectively. Then the graphene yield is:1
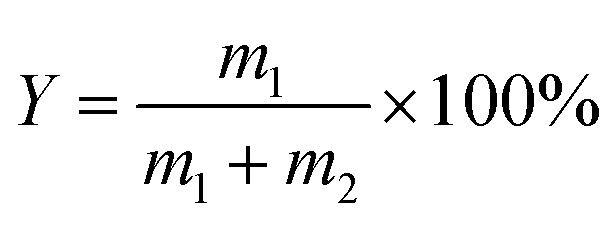
In the formula: *m*_1_ is the mass of the upper layer liquid; *m*_2_ is the mass of the lower layer precipitated.

#### 2.4.3. Performance analysis of EGF and MWEGF

Measured with a four-probe tester (RTS-9, Shenzhen Excellence Instrument Co., Ltd.). The surface temperature was measured with a thermal imaging thermometer (ST-8450, Suzhou Wanchuang Company). A vector network analyser (Rohde & Schwarz) was used to test the electromagnetic shielding effectiveness of EGF and MWEGF in the frequency range of 8.2–12.4 GHz.

## 3. Results and discussion

### 3.1. Optimization of EG preparation conditions

In general, there is a close relationship between its exfoliation quality and electrochemical exfoliation conditions. To this end, we explored the electrochemical preparation conditions in detail, taking the product's yield, conductivity, and defect results as the object of investigation. Firstly, NH_4_Cl, Na_2_SO_4_, (NH_4_)_2_SO_4_, K_2_SO_4_, (NH_4_)_2_HPO_4_, and Na_5_P_3_O_10_ were selected as electrolytes, and the electrolytes were screened under the conditions of electrolyte concentration 0.1 M, working voltage 10 V, pH 7, and exfoliate for 2 h.

**Fig. 2:**
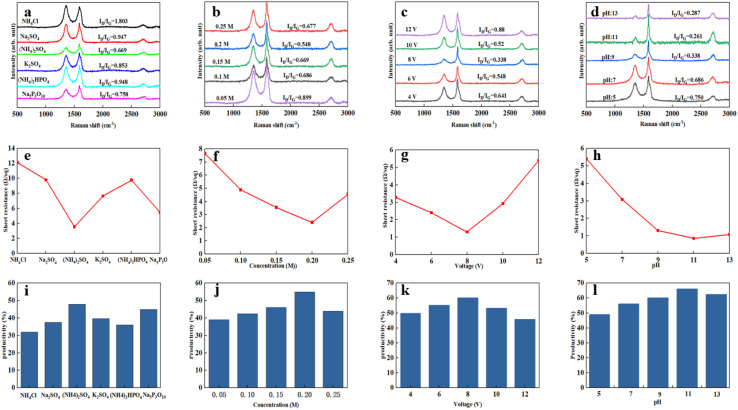
Raman diagram (a), square resistance (e) and yield (i) of graphene peeling from different electrolytes (NH_4_Cl, Na_2_SO_4_, (NH_4_)_2_SO_4_, K_2_SO_4_, (NH_4_)_2_HPO_4_, and Na_5_P_3_O_10_); Raman diagram (b), square resistance (f) and yield (j) of graphene peeling from different concentration (0.05 M, 0.10 M, 0.15 M, 0.20 M, 0.25 M); Raman diagram (c), square resistance (g) and yield (k) of graphene peeling under different voltage (4 V, 6 V, 8 V, 10 V, 12 V); Raman diagram (d), square resistance (h) and yield (l) of graphene peeling from pH (5, 7, 9, 11 and 13).

Raman spectroscopy has been widely used to evaluate graphene defects. The ratio of the signal intensities at 2D (2700 cm^−1^) and G (1590 cm^−1^) peaks confirm that graphene has a varying thickness. The characteristic peak at the D (1350 cm^−1^) band implies defect and disordered carbon. As shown in Fig. 2(a), the graphene prepared with (NH_4_)_2_SO_4_ as the electrolyte had an *I*_D_/*I*_G_ value of 0.669, indicating that the degree of graphitisation of the product was relatively low and showed fewer defects. At this time, its conductivity was excellent, the square resistance was only 3.55 Ω sq,^−1^ and the yield was 47.9%. When (NH_4_)_2_SO_4_ was used as the electrolyte, the graphene yield was higher than other electrolytes, and its conductivity was also the best. It was because the interlayer spacing of graphite was 0.335 nm, while the sulfate ion size was about 0.46 nm, which was similar to the interlayer spacing of graphite. So that it was easier to intercalate into the graphite interlayer than other ions. According to the previous reports,^22^ the electrolysis and co-intercalation of sulfate ions lead to producing gases such as SO_2_, O_2_, and H_2_. Efficient intercalation and gas eruption facilitate the separation of graphene sheets from adjacent graphene layers. The radius of ammonium ions is more significant than that of sodium ions and potassium ions. Partial intercalation can also be performed at the cathode to promote the expansion of the graphite electrode. After the electrolysis of water, the H^+^ generated by the electrolysis of water is reduced at the cathode to generate hydrogen to further promote the graphite electrode's exfoliation. Therefore, choosing ammonium sulfate as the electrolyte for electrochemical exfoliation is more appropriate.

Then, the effects of (NH_4_)_2_SO_4_ concentrations (0.05 M, 0.10 M, 0.15 M, 0.20 M and 0.25 M) on graphene's yield and conductivity were investigated under operating voltage conditions 10 V, pH 7, and stripping for 2 h. The results are shown in Fig. 2(b), (f) and (j). When the ammonium sulfate concentration was 0.2 M, the graphene yield was the highest at 55%. Its electrical conductivity was also the best, with a square resistance of 2.4 Ω sq^−1^, as shown in Fig. 2(f). But when the concentration increased to 0.25 M, the graphene yield decreased. With the increase of the ammonium sulfate concentration, the graphene production increased firstly and then dropped the time required for complete exfoliation also reduced continuously. This is because as the concentration increases, within the same processing time, more sulfate ions are intercalated between the graphite sheets, resulting in weakened interaction between the sheets, and more graphite could be exfoliated, which can effectively increase the hardness of graphene. When the concentration of ammonium sulfate is too high, the formation of hydroxyl radicals is suppressed due to the low water content. The graphite edge expansion and ion intercalation processes were expected to be relatively slow. Moreover, when the sulfate concentration was high, the degree of oxidation of the graphene rods was high, and the electrical conductivity decreased due to structural defects. Therefore, when the concentration of (NH_4_)_2_SO_4_ was 0.2 M, the *I*_D_/*I*_G_ of graphene was 0.548, and the degree of graphitization was low. Thus, the concentration of (NH_4_)_2_SO_4_ was chosen to be 0.2 M.

Secondly, the effects of (NH_4_)_2_SO_4_ electrolyte solutions with different voltages (4, 6, 8, 10 and 12 V) on the exfoliated graphene products under the conditions of electrolyte concentration of 0.2 M, pH 7, and stripping time of 2 h were further explored. The results are shown in Fig. 2(c), (g) and (k). As the voltage increases, the graphene yield increased first and then decreases. When the voltage increases, the driving force increased, and the diffusion rate of hydroxyl radicals and sulfate ions in the solution increases, which will make more ions embedded in the graphite layer. Hence, the output of graphene gradually increased with the rise in voltage. The conductivity was enhanced, as shown in Fig. 2(g). Since the graphite rod is made of compressed graphite particles, a large amount of gas generated had a potent force on the graphite interlayer when the voltage was too high. Since the sulfate ions had not yet been inserted into the layer, the severe gas impact caused the graphite particles to fall off before completely peeling off. At this time, the yield of graphene decreased, and the defects increased, leading to the product's poor conductivity. When the voltage was 8 V, the minimum defect *I*_D_/*I*_G_ = 0.338 was observed in the prepared graphene. Therefore, 8 V was selected as the appropriate voltage with better yield and conductive. The yield was 60% at this timed was 60%, and the square resistance was 1.3 Ω sq^−1^.

Finally, based on the previous exploration, we continued to explore the effect of (NH_4_)_2_SO_4_ electrolyte solutions with different pH values (5, 7, 9, 11, 13) on the exfoliated graphene products (Fig. 2(d), (h) and (l)). It was found that the interaction between hydrogen ions and sulfate ions leads to excessive oxidation of graphene under acidic conditions. When sodium hydroxide was added, under the action of electric field force, ammonium ions and hydroxyl groups in sodium hydroxide generated ammonia gas at the cathode further to promote the expansion and peeling of graphite rods, increasing the yield of graphene, cathodic attracting cation migration can prevent graphene from being excessively oxidised, maintains the integrity of sp^2^ the carbon ring structure, and enhance graphene's conductivity the pH was at set 11, the yield of the product was 66%, and the square resistance of the graphene sample was 1.6 Ω sq^−1^. But when the pH was greater than 11, the yield of graphene decreased, and the conductivity became poor. Because the high concentration of sodium hydroxide electrolyte affected the migration rate of anions. In the end, the surface of the graphite rod with severe expansion was over-expanded, accompanied by the peeling off of graphite particles. When the pH of the electrolyte was chosen as 11, the minimum *I*_D_/*I*_G_ of the prepared graphene defect was 0.261, exhibiting the low defect degree.

### 3.2. Characterization of EG

**Fig. 3:**
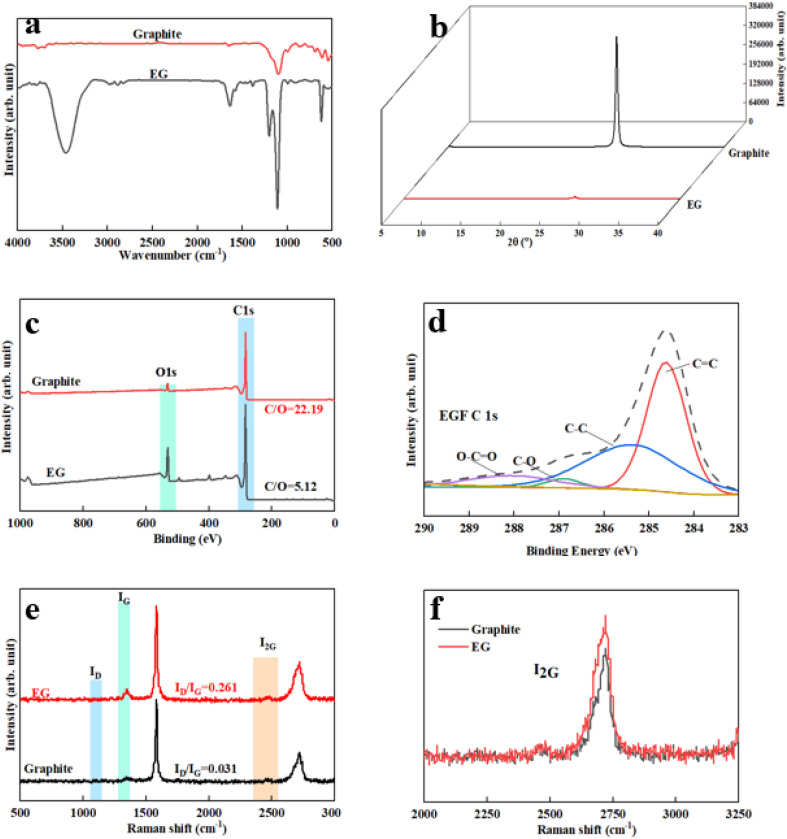
FT-IR spectra of EG and graphite (a). XRD diffraction patterns of EG and graphite (b). The XPS spectrum of EG and graphite (c). The C 1s XPS spectrum of EGF (d). Raman spectra of EG and graphite (e and f).

Above all, we explored in detail the microstructure and properties of the product samples of the conditions under optimal experimental conditions. Fig. 3 is the Fourier transform infrared spectrum of EG. New characteristic peaks appear at 3400 cm^−1^, 1620 cm^−1^, and 1200 cm^−1^, respectively, after graphite rods were electrochemically stripped. A broad and robust absorption peak occurred at 3400 cm^−1^, which was attributed to the hydroxyl-OH stretching vibration peak of EG forming hydrogen bonds with water molecules; the characteristic peak at 1620 cm^−1^ corresponds to the absorption of the C=C bond in the EG structure and the out-of-plane bending vibration absorption peak of –OH; the absorption peak at 1200 cm^−1^ corresponds to the stretching vibration of the epoxy group –C–O–C–. The characteristic peak at 1083 cm^−1^ is the stretching vibration of –C–OH. Theoretically, EG contains some –OH and –C–O–C– because EG may only be partially oxidized.^22,23^

Secondly, Fig. 3(c) is the XPS broad spectrum of EG. The C/O value of EG was 5.12, which is lower than the C/O = 22.19 of the raw graphite. In addition, the ultra-low oxidation degree can effectively guarantee mild conductivity and show good dispersion stability. According to relevant literature, this value was between reduced graphene oxide RGO (≈10–16) and GO (1–3).^24^ Generally speaking, a low degree of oxidation means that the damage to graphene conjugation was soft, so the integrity of the graphene structure can be guaranteed. Fig. 3(d) is the C-fitting gram of EG atoms. It can be seen that there were four fitting curves for C of EG, and these four fitting peaks correspond to C=C (284. V), C–OH (285.5 eV), C=O (287.6 eV), and C(O)–O (290.1 eV). It further showed that the EG surface contained a small amount of hydroxyl and carboxyl groups. In addition, we compared the XRD patterns of multilayer graphene and initial graphite. It can be seen that when the 2*θ* is about 26.42°, the characteristic peak of the graphite (002) crystal plane was sharp, the degree of crystallinity was high, and the interlayer spacing was about 0.335 nm. Compared with the initial characteristic peak of graphite, the intensity of the characteristic peak of the (002) crystal plane of the multilayer graphene exfoliated by the electrochemical method was weakened, indicating that the graphite expanded and exfoliated, and the degree of crystallization was reduced. The peak width of the characteristic peak increased, indicating that the disorder of the graphite sheet increased. At the same time, compared with pristine graphite rods, the (002) peak position of the EG peak did not shift to a small angle, which means that the interlayer spacing remains unchanged, and the exfoliated graphene product may only contain a small number of functional groups.^25^

**Fig. 4:**
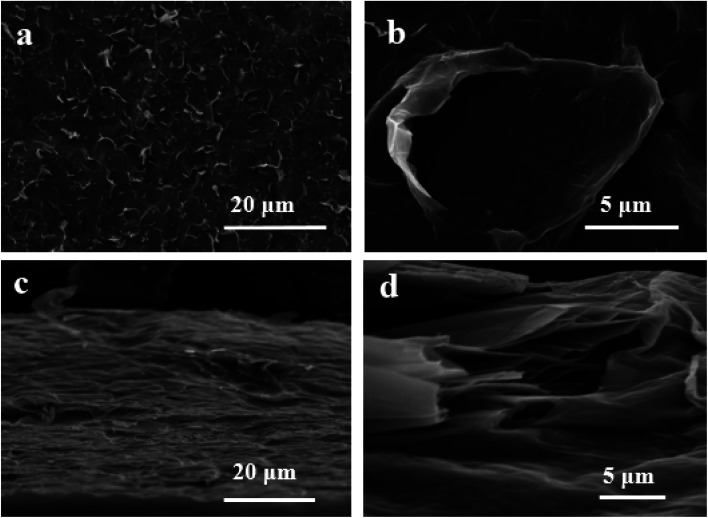
SEM images of EG surfaces (a and b) and longitudinal sections (c and d).

**Fig. 5:**
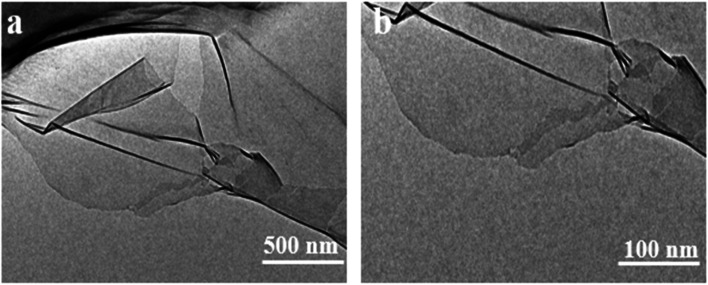
TEM images of EG (a and b).

**Fig. 6:**
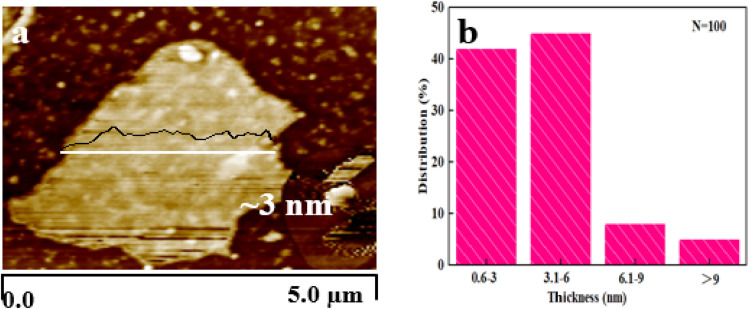
AFM images of EG (a). Thickness distribution of EG (b).

Furthermore, the Raman spectrum of pristine graphite presented three characteristic peaks: a very weak D peak at 1350 cm^−1^ caused by defects, a strong G peak at 1580 cm^−1^, and a 2D peak at 2708 cm^−1^ elevation. The value of *I*_D_/*I*_G_ was only 0.057. This indicates a lower defect level in pristine graphite, as shown in Fig. 3(e). The electrochemically exfoliated graphene samples were observed to have a slight increase in the intensity of the D peak, mainly due to the sp^3^ hybridisation of carbon during intercalation and ultrasonication, resulting in the attachment of oxygen-containing functional groups to the graphene edge, introducing a small amount of oxygen-containing functional groups.^26–28^ The *I*_D_/*I*_G_ value was 0.261, which indicated that the graphene prepared from electrochemical exfoliation had a higher quality, which was much lower than that of thermally reduced or chemically reduced graphene oxide (*I*_D_/*I*_G_ ≈ 1.1–1.5), which further illustrated that graphene process introduced only a small number of structural defects. The G peak was caused by the stretching vibration between carbon atoms of the sp^2^ structure, which reflects the crystallinity of the graphite. The intensity of the G peak in the figure was more significant, indicating that EG had a complete crystal structure. Compared with graphite rods, the 2D peak of electrochemically exfoliated graphene had a higher degree of symmetry, increased intensity, and a significant left shift in the peak position, confirming that the number of layers decreases when graphite rods are exfoliated into graphene, as shown in Fig. 3(f). Fig. 4 is an SEM image of graphene prepared by electrochemical exfoliation. It can be seen from Fig. 4(a) that the distribution of EG sheets is relatively uniform; Fig. 4(b) it can be seen that the size of EG sheets is mostly around 5–10 μm, which is different from that of chemical graphene oxide (∼40 μm) and mechanically exfoliated graphene (∼2 μm) are similar in size. Notably, the graphene prepared by the electrochemical exfoliation method was smoother and had no apparent wrinkle structure in the plane, only slightly wrinkled at the edge. The longitudinal section of EGF was observed, as shown in Fig. 3(c). The longitudinal section presents a compact and orderly layered structure. Fig. 3(d) is a locally enlarged view of the EGF longitudinal section. EGF surface gap, internal hole structure and residual water molecules reduce the contact between graphene sheets, but the stacking of graphene sheets in the layered structure becomes more orderly. Fig. 5 is the TEM diagram measured by dropping EG dispersion on a copper mesh microgrid. Fig. 5(a) shows the EG image at low resolution. It can be seen that the contour of EG is thin and has some wrinkles; the analysis shows that graphene is a strict two-dimensional atomic crystal with tremendous surface energy, which is easy to produce micro distortion. The conversion of the surface from two-dimensional to three-dimensional reduces the surface energy, reduces the free power of the system, and increases stability. However, graphene can still be spread with good flatness, which indicates that after electrochemical intercalation treatment, the force between the layers is weakened, and further shear ultrasound can better separate the layers. It can be seen from the edge that graphene prepared by electrochemical stripping is an oligo-layer structure (Fig. 5(b)). AFM accurately measured the size of the graphene. Fig. 6 is the AFM diagram of EG obtained. From the curve distribution of scanning in Fig. 6(a), the lateral dimension of EG is about 5 μm. Nanosheets with an average thickness were about 3 nm. Fig. 6(b) The statistical analysis of the flake thickness measured by AFM showed that 40% of the peeled EG flakes were <4 nm thick, further proving the high quality of graphene products.

The mechanism for the electrochemical preparation of graphene was generally assumed to be the result of the synergistic effect of ion intercalation and gas exfoliation. The graphite rod in the electrolyte is applied with a voltage, causing the water molecules at the cathode to be consumed. The electrolysis of water usually produces hydrogen ions and hydroxyl radicals. It is worth noting that hydroxyl radicals act as strong nucleophiles in the electrolyte. During constant voltage electrolysis, cations and anions in the electrolyte will move to the cathode and anode, respectively. The edge positions and grain boundaries of graphite were eroded first so that the graphite edges became loose; hydroxyl radicals generated by electrolysis of water acted as strong nucleophiles and continued to attack the sp^2^ hybridised carbon atoms at graphite edges and grain boundaries and, at the same time made two adjacent carbon atoms be hydroxylated. Subsequently, the two hydroxyl groups can interact with each other to form an epoxy ring. Likewise, they can also dissociate to form two carbonyl groups. At this stage, water molecules and sulfate anions co-intercalate to promote the expansion of anode graphite; the partial reduction of sulfate ions and the autooxidation of water generate gas that strips the graphite disperses it into solution. During this reaction process, the electrolysis of water and the intercalation of ions are interactive and synergistically promote graphite exfoliation. In addition to the oxidation reaction of graphite, other reactions take place, including releasing CO_2_ and O_2_ through the response. The generation of these gases can exert additional forces on the graphitic layers, separating the weakly bonded graphitic layers from each other. At the same time, under the action of the electric field force, the ammonium ion and the hydroxyl group in the sodium hydroxide generate ammonia gas at the cathode, further promoting the graphite rod's expansion and peeling.^17^

**Fig. 7:**
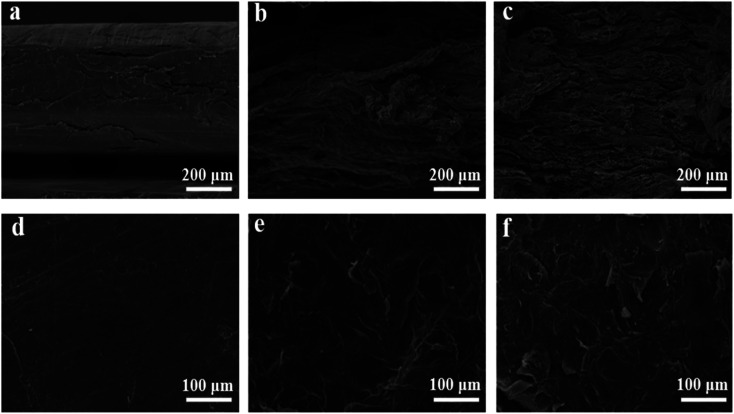
SEM of graphite electrode with different stripping times 1 min (a and d), 30 min (b and e), 60 min (c and f).

To further explore the specific process and microscopic mechanism of electrochemical oxidation exfoliation of graphene, the above mechanism hypothesis was verified by observing the morphology of graphite rods during the exfoliation process. A constant bias voltage was applied to the graphite electrode, and the morphology changes of the graphite rod after peeling off for 0–60 min were detected by SEM, as shown in Fig. 7(a)–(f). Fig. 7(a) and (d) are the longitudinal and horizontal surface SEM images of unexfoliated graphite rods.

**Fig. 8:**
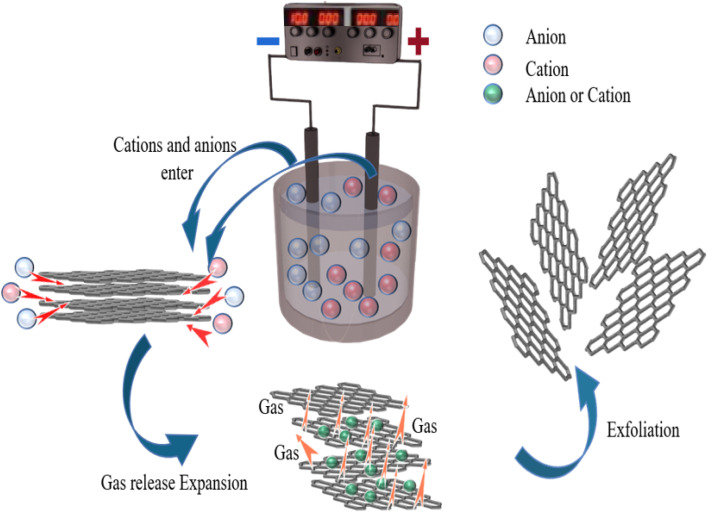
Mechanism diagram of double electrode electrochemical exfoliating graphene.

The surface of the graphite rod was flat and had a specific lustre. In the longitudinal direction, it can be seen that the layers were closely combined, showing a sheet or plate structure, and the edges were not warped. When a voltage is applied, graphite's surface and edge morphology change drastically; the graphite rod begins to expand longitudinally. At the same time, the cracks in the graphite layer increase, increasing the interlayer distance. There are many pores inside, forming a loose and porous structure, which is conducive to the further exfoliation of graphene, and many wrinkles are formed on the surface, as shown in Fig. 7(b) and (e). When exfoliated for 60 min, many graphene flakes were exfoliated and dispersed into the electrolyte solution. The longitudinal expansion of graphite rods was almost 10 times, as shown in Fig. 7(c). Furthermore, the corrugated network on the graphite surface was identified in the SEM images. This may be due to the graphitic layer's expansion due to the visible gas's evolution, as shown in Fig. 7(f). It's proved that the electrochemical exfoliation method to prepare graphene proceeds, as shown in Fig. 8. The edges and grain boundaries of the graphite electrode become loose, which is conducive to ion intercalation and leads to the exfoliation of graphene sheets.

**Fig. 9:**
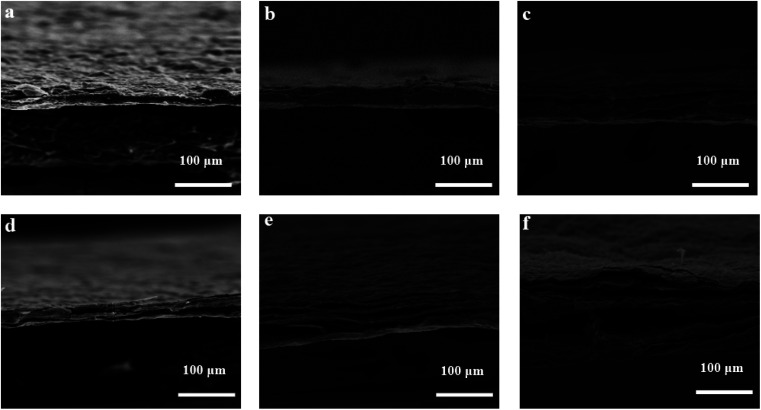
SEM of EGF and MWEGF longitudinal section with different loads of EGF-30 mg (a), EGF-50 mg (b), EGF-70 mg (c), MWEGF-30 mg (d), MWEGF-50 mg (e), MWEGF-70 mg (f).

Microwave reduction has attracted extensive attention in removing graphene due to its efficient and rapid heating method.^29^ In this experiment, we designed a set of quartz-based fixtures to ensure the integrity of the graphene film during reduction. Because we found that the resulting self-supporting graphene films were often broken or incomplete if there was no quartz cover to hold them in place, this was because the graphene film may impact the graphene stack due to uneven heating or the generation of a large amount of gas under microwave radiation. As shown in Fig. 1, the graphene film surface showed apparent voids and incomplete structure without volume confinement after radiation reduction. Fig. 9 is the cross-sectional SEM scanning diagrams of EGF and MWEGF, wherein the charts are the cross-sectional SEM scanning diagrams of loading amounts of 30, 50, and 70 mg, respectively. With the increase of graphene loading, the thickness of EGF and MWEGF increased, reaching 51.87 μm and 106.9 μm, respectively. The overall consistency of MWEGF was thicker than that of EGF, which may be caused to the formation and release of bubbles on the membrane surface during the removal of oxygen-containing functional groups in the microwave reduction process.^30^ Thereby changing the flat morphology of the EGF membrane to a raised foam shape with increased thickness. Moreover, the interlayer spacing inside the film will increase compared to before microwave reduction.

### 3.3. Comparative analysis of structures of EGF and MWEGF

**Fig. 10:**
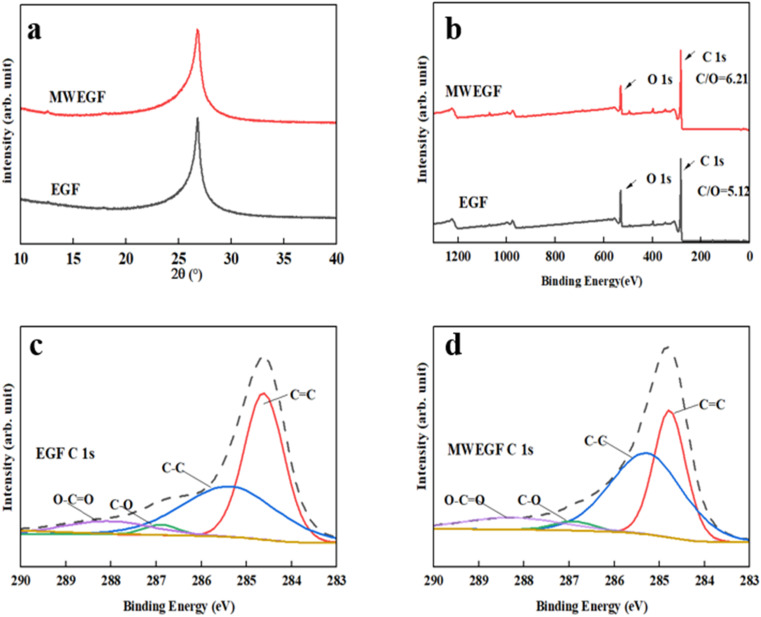
XRD of EGF and MWEGF (a), XPS spectrum of EGF and MWEGF (b), C 1s spectrum analysis diagram of EGF (c) and MWEGF (d).

It can be seen from the figure that the overall structure of the graphene film had little change before and after microwave reduction. When 2*θ* was about 26.42°, the crystallisation degree of EGF and MWEGF decreased. Compared with EGF, the half-width of MWEGF was more comprehensive, and the disorder of graphite sheets was further enhanced. Since the full width at half maximum was inversely proportional to the grain size, the smaller the grain diameter, the larger the full width at half maximum. It is indicated that the grain size of MWEGF is smaller than EGF's. Fig. 10 is the C 1s XPS elemental spectrum of EGF and MWEGF. From the C 1s XPS spectrum, it can be found that both EGF and MWEGF exhibit high C/O ratios, 5.19 and 6.2, respectively. Especially the C/O percentage of MWEGF was much larger than the chemically synthesised graphene oxide film. Fig. 10(c) and (d) are the C 1s fitting diagrams of EGF and MWEGF, respectively. By comparison, it can be found that the peak of C–C (285.3 eV) of MWEGF was significantly higher than that of EGF. Combined with the full spectrum of C 1s, it can be seen that the content of carbon elements had increased, indicating that graphene films were successfully reduced under the action of microwaves.

### 3.4. Performance analysis of EGF and MWEGF

#### 3.4.1. Electrical performance analysis of EGF and MWEGF

**Fig. 11:**
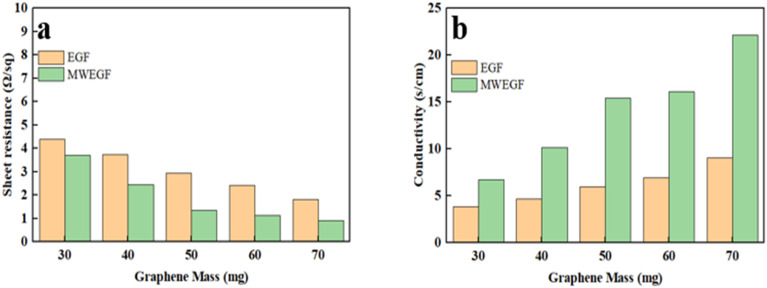
Square resistance (a) and conductivity (b) of EGF and MWEGF with EG loading.

Fig. 11(a) is a graph showing the change of square resistance with the mass of EGF and MWEGF. As the group of graphene increases, the resistance of EGF and MWEGF gradually decreases. When the mass reached 70 mg, the square resistance of EGF was 1.82 Ω sq^−1^, and the conductivity was 9.09 S cm^−1^, which showed good electrical conductivity. Compared with EGF of the same quality, the conductivity and square resistance of MWEGF obtained after microwave reduction have been improved. When the mass of graphene reaches 70 mg, the square resistance is 0.92 Ω sq^−1^, and the highest conductivity can reach 22.12 S cm^−1^. EG was subjected to high temperatures reduction during the microwave reduction process, so a small amount of oxygen-containing functional groups carried by EG were sintered.^31^ The degree of oxidation of EG prepared was reduced by electrochemical exfoliation, and the electrical conductivity was improved.

#### 3.4.2. Thermal conductivity analysis of EGF and MWEGF

**Fig. 12:**
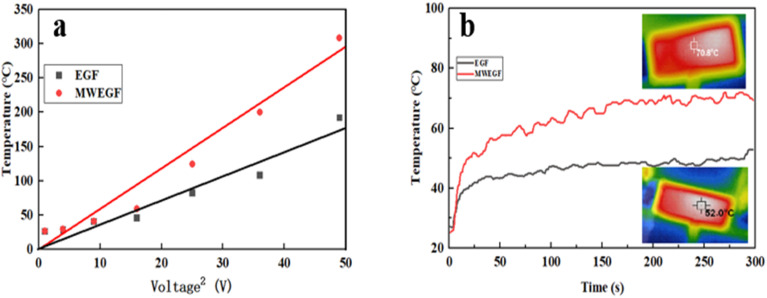
Linear fitting diagram of saturation temperature to the voltage of EGF and MWEGF (a), time–temperature curve at 4 V voltage (b).

Fig. 12(a) shows the data variation relationship between the saturation temperature and voltage of EGF and MWEGF under the supply voltage of 1 to 7 V. Under the low voltage condition of 1–3 V, EGF and MWEGF increased with the voltage at room temperature of 22 °C, the surface temperature rose relatively slowly, and their surface temperature difference was similar. At 4–7 V, their surface temperature differences start to be significant. As the voltage was increased to 4 V, the surface temperature of EGF and MWEGF began to rise significantly, and the temperature difference between them gradually widened. When the voltage reached 7 V, the surface temperature of EGF and MWEGF reached the highest value, 180 °C and 320 °C, respectively. It is believed that the current passing through EGF and MWEGF can still generate large Joule heat even at low supply voltage due to the high conductivity of EGF and MWEGF. Fig. 12(b) shows the time–temperature performance comparison of EGF and MWEGF at a low supply voltage of 4 V, heating from room temperature of 22 °C, EGF can reach a stable temperature in 43 s. However, MWEGF reached a steady temperature of 68 s, and the surface temperature of the film fluctuated considerably during the heating process. Combined with the thermal conductivity of EGF and MWEGF in Table 1 and the graph analysis, this may be due to the increase in the internal layer spacing of MWEGF obtained after microwave reduction, which leads to a decrease in the degree of conductive network connection and thermal conductivity of MWEGF. However, the high conductivity of MWEFP makes its electric heating performance better than that of EGF. Due to the Joule heat generated by EGF with the high response, the heating process was stable compared with MWEGF. The surface temperature of EGF can be easily customised by changing the supplied voltage for intelligent, flexible wearables to ensure the safety of the human body.^32^ MWEGF is more suitable for thermal insulation because of its relatively low thermal conductivity and high joule electric heating performance.^31,33^

**Table 1:** Thermal conductivity of EGF and MWEGF

	50 mg-1	50 mg-2	50 mg-3
EGF	0.1410 W m^−1^ K^−1^	0.1342 W m^−1^ K^−1^	0.1340 W m^−1^ K^−1^
MWEGF	0.05819 W m^−1^ K^−1^	0.05708 W m^−1^ K^−1^	0.05685 W m^−1^ K^−1^

#### 3.4.3. Electromagnetic shielding effectiveness analysis of EGF and MWEGF

**Fig. 13:**
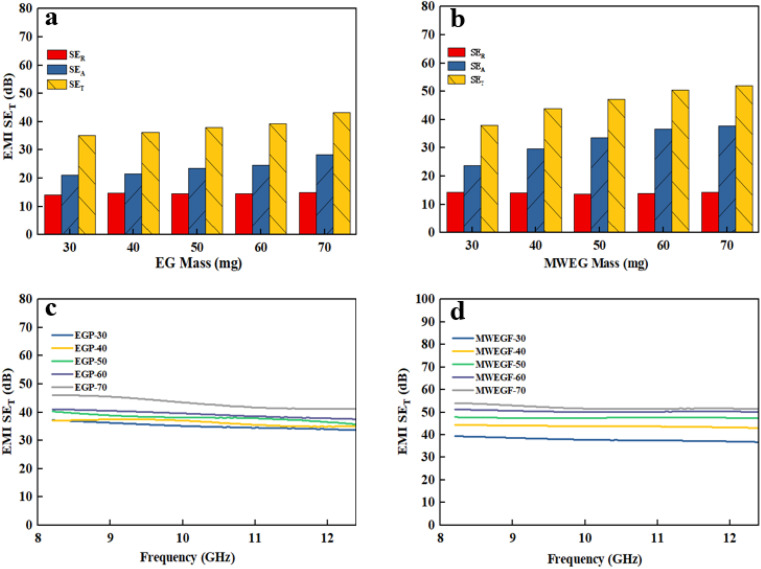
Electromagnetic shielding effectiveness diagram of EGF (a and c) and MWEGF (b and d) at different loading.

Electronic equipment's operation will generate adverse electromagnetic interference (EMI) to the outside world.^34,35^ It affects the average regular of nearby electronic equipment and increases the risk of headaches, depression, immune deficiency and other diseases for relevant staff.^36–38^ Therefore, there is an urgent need for efficient EMI shielding materials to attenuate electromagnetic waves to protect the regular operation of electronic equipment and human health. Developing graphene-based high-performance EMI shielding materials has become a research hotspot in this context. Fig. 13 shows the variation in electromagnetic shielding effectiveness of EGF and MWEGF in the frequency range of 8.2–12.4 GHz under different mass loadings. SE evaluates the attenuation performance of shielding materials; absorption loss (SEA), reflection loss (SER), and multiple reflection loss (SEM) constitute the total electromagnetic shielding effectiveness (SET). Since the SET of EGF and MWEGF are greater than 15 dB, the SE_M_ is negligible. The calculation formulas of SE_T_, SE_A_ and SE_R_ are as follows:SE_T_ = −10 log *T*SE_R_ = −10 log(1 − *R*)
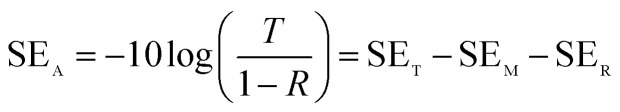
In the formula: SE_T_, SE_A_, SE_R_, and SE_M_, respectively, represent the total electromagnetic shielding effectiveness, electromagnetic wave absorption loss, electromagnetic wave reflection loss and multiple reflection loss of electromagnetic waves; *T* and *R* represent the power coefficients of transmission and reflection, respectively.^37,39^

**Fig. 14:**
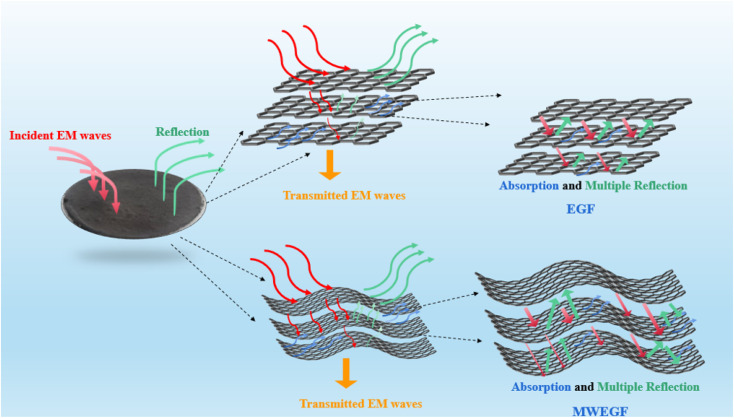
MWEGF electromagnetic shielding mechanism diagram.

It is the electromagnetic shielding effectiveness diagram of EGF and MWEGF with different masses in the 8.2–12.4 GHz frequency range. The attenuation performance of the shielding material is evaluated by SE, as shown in the Fig. 13(a) and (b). As the mass of graphene increases, the total electromagnetic shielding effectiveness of EGF and MWEGF gradually increases. When the EG mass reached 70 mg, EGF's actual electromagnetic shielding effectiveness was 43 dB, and the entire electromagnetic shielding effectiveness of MWEGF was 52 dB, far exceeding the commercial requirement value of 20 dB. Compared with the published literature, the performance of MWEGF samples in this paper was competitive under the constraints of simple, low-cost and environmentally friendly preparation strategies (Table 2). It can be seen that the electromagnetic absorption ability (SE_A_) of EGF and MWEGF far exceeded the electromagnetic reflection ability (SE_R_). The analysis showed MWEGF had better conductivity and larger internal layer spacing than EGF. When the electromagnetic wave passes through the MWEGF film, it is first consumed by the reflection part of the surface conductive network. Then when the electromagnetic wave enters the inside of the MWEGF, it will continuously undergo reflection loss and absorption consumption between the graphene sheets. It is better than EGF, with a relatively compact internal structure and relatively poor conductivity. In addition, the irregular interlayer structure of MWEGF increased the diversity of reflection direction, causing the reflection path to grow. The specific electromagnetic shielding mechanism of MWEGF is shown in Fig. 14.

**Table 2:** Summary of literature on electromagnetic interference shielding with different reduction method

Chemical composition	Method	Reduction time	Thickness	EMI SET	Ref.
RGO	Microwave reduction	1 min	106.9 μm	52.3 dB	This work
Polyaromatic ether sulfone with carbon (PES-C), RGO	Chemical reduction by HI, then microwave reduction	8 h	122 μm	46.73 dB	40
GO, copper phthalocyanine (CuPc)	Thermal reduction (800 °C)	>24 h	470 μm	55.2 dB	41
GO	Thermal reduction (1000 °C)	>4 h	50 μm	45–54.3 dB	42
GO	Chemical reduction, thermal reduction	>15 h	77 μm	50 dB	43
VGS, PI	CVD	>3 h	151 μm	31.37 dB	44

## 4. Conclusion

This paper uses conductivity, yield and structure as evaluation factors for the first time to systematically discuss the effects of electrolyte type, concentration, voltage, and pH on the yield and conductivity of graphene prepared by electrochemical exfoliation. Without introducing any organic solvent and other auxiliary equipment, the best conditions for the preparation of graphene by double-electrode electrochemical exfoliation are as follows: the electrolyte is ammonium sulfate, the concentration is 0.2 M, the voltage is 8 V, and the pH is 11. The square resistance of the product sample was 1.6 Ω sq^−1^, and the yield was 65%, *I*_D_/*I*_G_ = 0.261 meanwhile, the structural defects were minor. The results show that the C/O value of EG was 5.19, the oxidation degree of the sample was low, the defect degree was small, and the position where the intensity of the characteristic peak of (002) crystal plane weakens remains unchanged. In addition, the C/O value of EG by microwave reduction could be achieved at 6.2, indicating that the graphene prepared by this method had a relatively complete structure and had a good crystal and graphitised form. The analysis results of EGF and MWEGF electrical, thermal and electromagnetic shielding properties show that MWEGF has more electrical conductivity than EGF. Still, EGF has more vital controllable thermal conductivity, possibly due to EGF's tighter internal sheet structure and interlayer. The conductive network is more complete. In addition, due to the high conductivity and irregular and complex interlayer structure of EMWEGF, it shows excellent performance in electromagnetic shielding, with a shielding coefficient of 53 dB, and has good application prospects in electromagnetic pollution protection and stealth.

## Conflicts of interest

The authors declare that they have no known competing financial interests or personal relationships that could have appeared to influence the work reported in this paper.

## Supplementary Material
